# Dental and maxillomandibular incidental findings in panoramic radiography among individuals with mucopolysaccharidosis: a cross-sectional study

**DOI:** 10.1590/1678-7757-2020-0978

**Published:** 2021-04-14

**Authors:** Natália Cristina Ruy CARNEIRO, Lucas Guimarães ABREU, Roselaine Moreira Coelho MILAGRES, Tania Mara Pimenta AMARAL, Carlos FLORES-MIR, Isabela Almeida PORDEUS, Ana Cristina BORGES-OLIVEIRA

**Affiliations:** 1 Universidade Federal de Minas Gerais Faculdade de Odontologia Departamento de Saúde Bucal da Criança e do Adolescente Belo Horizonte Brasil Universidade Federal de Minas Gerais, Faculdade de Odontologia, Departamento de Saúde Bucal da Criança e do Adolescente, Belo Horizonte, Brasil.; 2 Universidade Federal de Minas Gerais Faculdade de Odontologia Departamento de Clínica, Patologia e Cirurgia Belo Horizonte Brasil Universidade Federal de Minas Gerais, Faculdade de Odontologia, Departamento de Clínica, Patologia e Cirurgia, Belo Horizonte, Brasil.; 3 University of Alberta School of Dentistry Edmonton Canada University of Alberta, School of Dentistry, Division of Orthodontics, Edmonton, Canada; 4 Universidade Federal de Minas Gerais Faculdade de Odontologia Departamento de Odontologia Social e Preventiva Belo Horizonte Brasil Universidade Federal de Minas Gerais, Faculdade de Odontologia, Departamento de Odontologia Social e Preventiva, Belo Horizonte, Brasil.

**Keywords:** Mucopolysaccharidosis, Rare diseases, Dental care for the disabled, Tooth abnormalities

## Abstract

Mucopolysaccharidosis (MPS) is a group of rare and inherited metabolic disorders caused by the accumulation of macromolecule glycosaminoglycans inside lysosomes. Affected individuals may have dental and craniofacial tissue alterations, facilitating the development of several oral diseases.

**Objectives:**

To assess, with panoramic radiographic images, the frequency of dental and maxillomandibular incidental findings among MPS individuals and compare them with non-MPS individuals.

**Methodology:**

A cross-sectional study evaluating a sample of 14 MPS individuals and 28 non-MPS individuals aged from 5 to 26 years was carried out. They were matched for sex and age on a 2:1 proportion. Panoramic radiographs were assessed for the presence/absence of the following dental and maxillomandibular alterations: dental anomalies of number (hypodontia/dental agenesis, supernumerary teeth); anomalies of form (microdontia, macrodontia, conoid teeth, taurodontism, and root dilaceration); anomalies of position (impacted tooth, inverted tooth, tooth migration, partially bony teeth, complete bony teeth); periapical alterations (furcation lesion, circumscribed bone rarefaction); other alterations (radiolucent bone lesions, radiopaque bone lesions, radiopacity in the maxillary sinus, condylar hypoplasia). Differences between groups were tested by the Fisher’s exact test and chi-square test (p<0.05).

**Results:**

For intrarater agreement, Kappa values were 0.76 to 0.85. The presence of supernumerary teeth (p=0.003); conoid teeth (p=0.009); taurodontism (p<0.001); impacted teeth (p<0.001); partial bony teeth (p=0.040); complete bony teeth (p=0.013); and root dilaceration (p=0.047) were statistically more frequent in MPS individuals compared to non-MPS individuals. Bone rarefaction/furcation lesions (p=0.032), condylar hypoplasia (p<0.001), radiolucent bone lesions (p=0.001), and dentigerous cysts (p=0.002) were also more frequent in MPS individuals.

**Conclusion:**

The presence of specific oral manifestations is more common in MPS individuals than non-MPS individuals.

## Introduction

Mucopolysaccharidosis (MPS) are a group of inherited lysosomal storage diseases occurring due to a deficiency in some enzymes responsible for glycosaminoglycans (GAGs) degradation. The non-degradation of GAGs leads to their accumulation in cells and tissues, causing progressive accumulation and cell dysfunction.^1^There are seven types of MPS (I, II, III, IV, VI, VII, and IX), and the literature describes the overall prevalence of MPS ranging from 1.04 to 4.8/100.000 births.^2^

The nature of MPS is progressive, leading to several systemic manifestations that vary according to type and severity of the diseases. The most common systemic features observed in MPS individuals are the presence of inguinal and umbilical hernias, upper respiratory infections, corneal clouding, skeletal dysplasia, growth impairment, limited joint motion, and behavioral anomalies.^3,4^

Oral and craniofacial manifestations are also frequent in MPS individuals. The literature describes the presence of macroglossia, malocclusion traits (i.e., anterior open bite), delayed tooth eruption, cystic lesions, and condylar defects in individuals with MPS as typical.^5-8^ MPS individuals are also a group with a higher vulnerability to dental caries, therefore, requiring better guidance and access to preventive oral health programs.^9,10^

It has been stated in the literature that the presence of dental and/or maxillomandibular anomalies can be considered a hampering factor for some dental treatments, such as root canal therapy, or it may also cause pain, sensitivity or aesthetic problems to patients.^11^ Therefore, identifying such problems, mainly in individuals with disabilities, can minimize associated clinical consequences and enhance patients’ quality of life. Regarding to analyses of oral manifestations in individuals with MPS, there are few studies with adequate methodological quality. Most studies are case series that do not have a comparison group to provide clinicians with information on what they might expect when dealing with MPS individuals.^5-8^

Thus, this study evaluates, based on panoramic radiographic images, the frequency of dental and maxillomandibular incidental findings among individuals with MPS and comparing them with matched non-MPS individuals.

## Methodology

This article is in accordance with The Strengthening the Reporting of Observational Studies in Epidemiology (STROBE) statement.^12^

The Research Ethics Committee of the Universidade Federal de Minas Gerais approved this article (protocol No. 01480212.4.0000.5149). Parents/caregivers and individuals with and without MPS willing to participate voluntarily signed an informed permission or consent form.

A cross-sectional study was carried out with a sample of MPS individuals aged from 5 to 26 years matched with non-MPS individuals. MPS individuals were selected in two hospitals (Southeastern Brazil). There were 29 MPS individuals registered at the two hospitals. These individuals were assessed regarding eligibility. These hospitals are referral centers for the care of individuals with such condition. MPS can be classified in MPS type I, II, III, IV, VI, VII, and IX^2^ and its classification is carried out with laboratory exams, according to the enzyme involved in the degradation pathway of a specific GAG. In this study, the type of MPS was recorded by mother’s knowledge of a full diagnosis.

Non-MPS individuals were selected in the outpatient clinics of the same two hospitals. They were individuals without any systemic conditions or syndromes, just applying for an annual health care visit. MPS and non-MPS individuals were individually matched for sex and age on a 2:1 ratio. Individuals with previous orthodontic treatment history and those with uncooperative behavior during the radiographic examination were excluded from the study. Data collection occurred from January/2015 to December/2017.

Panoramic radiographic examination was performed as part of a comprehensive oral review. Presence or absence of the following dental and maxillomandibular alterations were considered in that examination:^13^ dental anomalies of number (hypodontia/dental agenesis, supernumerary teeth); anomalies of form (microdontia, macrodontia, conoid teeth, taurodontism and root dilaceration); anomalies of position (impacted teeth, inverted teeth, tooth migration, partial bony teeth, complete bony teeth); periapical alterations (furcation lesion, circumscribed bone rarefaction); other alterations (radiolucent bone lesions, radiopaque bone lesions, radiopacity in the maxillary sinus, condylar hypoplasia).

A single-blinded radiologist with over 10 years of experience performed the interpretation of the panoramic radiographs. The rater was previously calibrated for data collection. The panoramic radiographs of 15 non-MPS individuals were examined, for the calibration process. They were re-examined two weeks later for the intra-rater agreement estimation. Those radiographs were not included in the main study.

Before the main study, a pilot study was also performed. The sample was composed of five MPS individuals and five non-MPS individuals. The results indicated that changes in the methodological procedures were deemed unnecessary.

Data analyses were conducted using the Statistical Package for Social Sciences (SPSS for Windows, version 21.0, SPSS IBM Corp., Armonk, N.Y., USA). Descriptive and bivariate analyses were carried out. The Fisher’s exact test and chi-square (X^2^) test were used to compare MPS and non-MPS participants concerning the panoramic radiographic findings (*p*<0.05).

## Results

For the intra-rater agreement, Kappa values obtained ranging from 0.76 to 0.85 were very good.^14^

Among the 29 MPS individuals who were assessed regarding eligibility, 14 were included and evaluated. In total, 15 MPS individuals were excluded due to the advanced conditions of their disease – that made proper head positioning unfeasible – lack of cooperation during examinations, and previous orthodontic treatment. The final sample comprised 42 individuals (14 with MPS and 28 without MPS) matched for age and sex. Most participants were male individuals (57.1%). The mean age of the 42 individuals was 13.9 years (±7.2).

**Figure 1:**
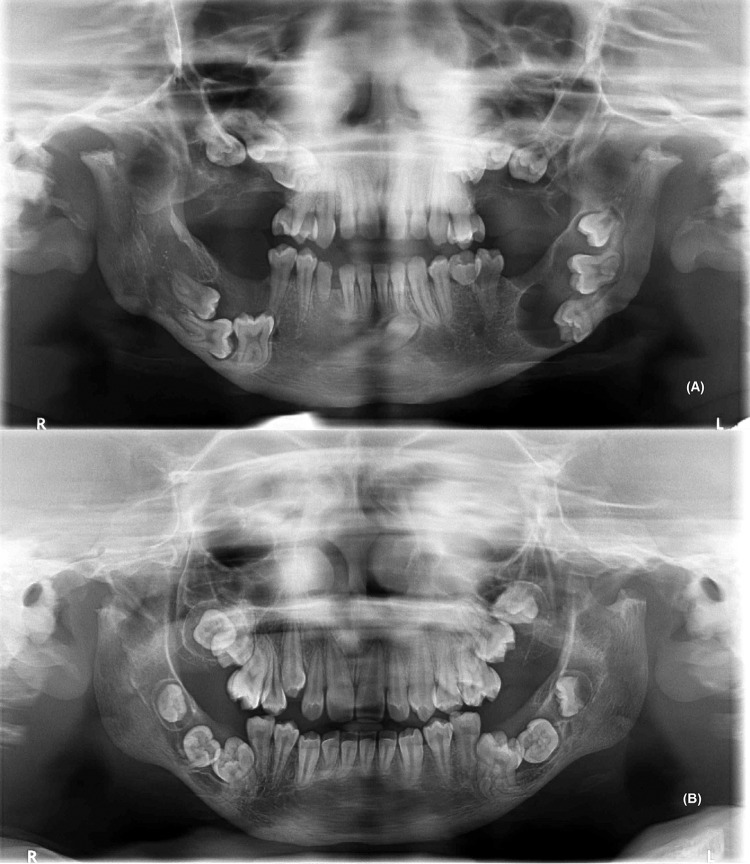
Panoramic radiographs of patients with MPS type VI, aged 19 (A) and 18 (B) years old, showing the presence of extensive maxillomandibular alterations

**Figure 2:**
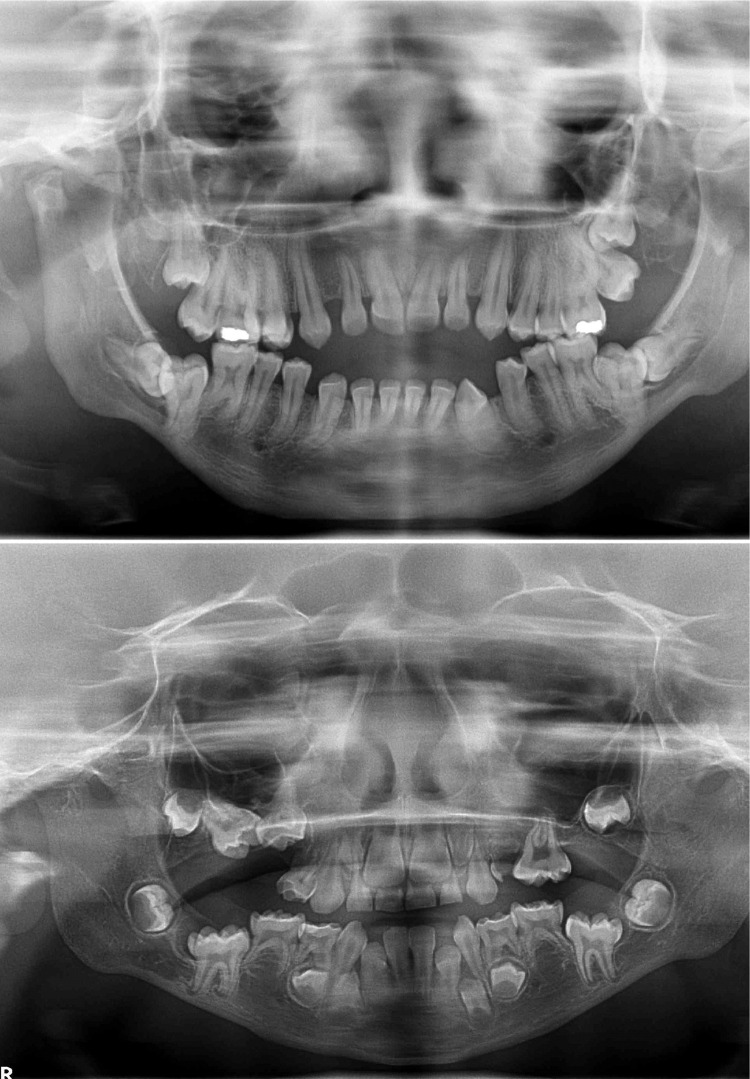
Panoramic radiographs of individuals with MPS type VI, aged 18 years old (A) and MPS type I, aged 6 years old (B), showing taurodontism, root dilaceration, tooth agenesis, and tooth impaction

Regarding the type of MPS, the individuals were classified as follows: MPS I (n=4), MPS II (n=2), MPS IV (n=1) and MPS VI (n=7). No individuals with MPS type III and IX were identified. Figure 1 and Figure 2 shows the panoramic radiographs of individuals with MPS and some identified dental alterations in the maxilla and mandible.

Table 1 shows the results of the comparison between MPS and non-MPS individuals concerning the presence of dental anomalies observed in panoramic radiographs. Some alterations were statistically more prevalent in MPS individuals compared to non-MPS individuals: supernumerary teeth (*p*=0.003); conoid teeth (p=0.009); taurodontism (*p*<0.001); impacted teeth (*p*<0.001); partial bony teeth (*p*=0.040); complete bony teeth (*p*=0.013); and root dilaceration (*p*=0.047)

**Table 1:** Comparative radiographic analysis of the presence of dental anomalies between MPS and non-MPS individuals (n=42)

	GROUP			
Dental abnormalities	MPS individuals	Non-MPS individuals	Odds ratio (Confidence Interval)	p Value
	n (%)	n (%)		
**ANOMALIES OF NUMBER**				
**Hypodontia (Dental Agenesis)**				0.647^a^
Presence	4 (28.6)	8 (28.6)	1.00 [0.24-4.13]	
Absence	10 (71.4)	20 (71.4)		
**Supernumerary teeth**				0.003^a^
Presence	7 (50.0)	2 (7.1)	3.66 [1.74-7.72]	
Absence	7 (50.0)	26 (92.9)		
**ANOMALIES OF FORM**				
**Microdontia**				0.100^a^
Presence	3 (21.4)	1 (3.6)	7.36 [0.68-78.71]	
Absence	11 (78.6)	27 (96.4)		
**Conoid teeth**				0.009^a^
Presence	4 (28.6)	0	3.80 [2.32-6.46]	
Absence	10 (71.4)	28 (100)		
**Taurodontism**				<0.001^a^
Presence	8 (57.1)	1 (3.6)	4.88 [2.28-10.45]	
Absence	6 (42.9)	27 (96.4)		
**ANOMALIES OF POSITION**				
**Impacted Teeth**				<0.001^a^
Presence	11 (78.6)	6 (21.4)	13.44 [2.81-64.20]	
Absence	3 (21.4)	22 (78.6)		
**Inverted teeth**				0.333^a^
Presence	1 (7.1)	0	3.15 [2.01-4.94]	
Absence	13 (92.9)	28 (100)		
**Migration**				
Presence	1 (7.1)	0		0.500^a^
Absence	13 (92.9)	28 (100)	3.15 [2.01-4.94]	
**Partial bony teeth**				0.040^b^
Presence	8 (57.1)	7 (46.7)	4.00 [1.02-15.59]	
Absence	6 (42.9)	21 (75)		
**Complete bony teeth**				0.013^b^
Presence	9 (64.3)	7(25.0)	5.4 [1.34-21.63]	
Absence	5 (35.7)	21 (75.0)		
**ROOT ALTERATION**				
**Dilaceration**				0.047^b^
Presence	9 (64.3)	9 (31.1)	3.8 [1.02-14.66]	

^a^Fisher’s exact test/ b Pearson Chi Square (5% significance level)

Table 2 shows the comparative analysis of the frequency of maxillomandibular alterations observed among MPS and non-MPS individuals. The prevalence of bone rarefaction/furcation lesions (*p*=0.032) and condylar hypoplasia (*p*<0.001) were significantly more frequent in MPS individuals. Regarding other maxillomandibular alterations, the presence of radiolucent bone lesions (*p*=0.001), dentigerous cysts (*p*=0.002) was also more frequent in MPS individuals. The presence of condylar hypoplasia was also observed in all MPS individuals, whereas in only one non-MPS individual, this alteration was observed (*p*<0.001).

**Table 2:** Comparative radiographic analysis of the presence of maxillomandibular alterations between MPS and non-MPS individuals (N=42)

	GROUP			
Maxillomandibular alterations	MPS individuals	Non-MPS individuals	Odds [Confidence Interval]	P Value
	n (%)	n (%)		
**PERIAPICAL ALTERATIONS**				
**Bone rarefaction (furcation lesions)**				0.032^a^
Presence	3 (21.4)	0	3.54 [2.14-5.85]	
Absence	11 (78.6)	28 (100)		
**Circumscribed bone rarefaction**				0.333^a^
Presence	1 (7.1)	0	3.15 [2.01-4.94]	
Absence	13 (92.9)	28 (100)		
**OTHER**				
**Radiolucent bone lesions**				0.001^a^
Presence	9 (64.3)	3 (10.7)	15.00 [2.96-75.91]	
Absence	5 (35.7)	25 (89.3)		
**Diagnostic hypothesis for radiolucent bone lesions**				
Dentigerous cyst	8 (57.1)	3 (10.7)	11.11 [2.24-54.94]	0.002^a^
Absence	6 (42.9)	25 (89.3)		
**Radiopacity in the maxillary sinus**				0.374^b^
Presence	7 (50.0)	10 (35.7)	1.80 [0.49-6.61]	
Absence	7 (50.0)	18 (64.3)		
**Condylar Hypoplasia**				<0.001^a^
Presence	14 (100)	1 (3.6)	0.06 [0.01-0.44]	
Absence	0	27 (96.4)		

^a^Fisher’s exact test/ bPearson Chi Square/ (5% significance level)

## Discussion

The presence of dental and maxillomandibular alterations can have far-reaching effects on an individual’s quality of life, causing pain, increased sensitivity, and esthetic issues.^11^ Significant differences between MPS and non-MPS individuals regarding radiographic findings were observed. MPS individuals presented higher frequencies of incidental findings.

MPS individuals face several systemic alterations during the disease progression. Those alterations have been described in the literature, and the management guidelines have been reported.^15^However, due to the rarity of the disease regarding dental alterations, there is a lack of in-depth knowledge concerning dental alterations among oral health practitioners.

Identification of oral manifestations in MPS individuals can help clinicians diagnose dental diseases earlier and also better support dental treatment planning. This process may minimize possible complications that could affect those individuals. Radical examinations, such as panoramic radiographs, are still considered a significant dental screening tool. Radiographic equipment is easy to use, provides cost-effective low-dose radiation, and it is used in epidemiological studies to evaluate the presence of dental and maxillomandibular anomalies.^16^

Among dental abnormalities, the most relevant radiographic manifestations found in MPS individuals compared to non-MPS individuals were supernumerary tooth, conoid teeth, taurodontism, and impacted teeth (partially and/or completed bony), and root dilaceration. The presence of dental abnormalities can lead to some issues in the function and development of the stomatognathic system, such as a delayed transition from deciduous to permanent dentition, impairment of occlusion, altered chewing and aesthetic issues. In a group of individuals already facing systemic disabilities, early identification and timely management of dental anomalies are recommended.

The findings of previous studies corroborate our results as they showed that dental anomalies are highly frequent among MPS individuals. Almeida-Barros, et al.^6^ (2018), identified the presence of supernumerary teeth in 23.5% of the 17 MPS individuals evaluated (only MPS type IV and VI). The literature also indicates that the frequency of taurodontism and impacted teeth is higher among MPS individuals. According to Sarmento, et al.^17^ (2015), 68.7% of the evaluated MPS individuals presented both alterations. Kantaputra, et al.^18^ (2013), showed that 53.8% of their sample had taurodontism.

The literature suggests that the constant accumulation of GAGs in cells is the main factor involved in developing orofacial alterations in MPS individuals. However, the extent and significance of this process are still uncertain.^19,20^ Oussoren, et al.^21^ (2011) described that GAGs might interfere with the regulatory process of growth using the function of bone morphogenetic proteins alteration. These proteins are multi-functional growth factors belonging to the transforming growth factor β superfamily. Consequently, the interaction of GAGs in the growth pathway can lead to tooth cytodifferentiation and supporting periodontal tissue defects.

Panoramic radiographs also revealed furcation lesions and radiolucent bone lesions (with 57.1% of likely dentigerous cysts) were the most frequent maxillomandibular alterations observed in most MPS individuals compared to their non-MPS peers. Other studies showed that dentigerous cysts are highly frequent in MPS individuals. Sarmento, et al.^17^ (2015) observed a 75.0% frequency of dentigerous cysts occurrence. Ballikaya, et al.^7^ (2018) observed that 83.3% of the 12 MPS individuals examined also had enlarged dental follicles. The cystic transformation of the dental follicle in MPS individuals may also be associated with GAGs accumulation.

In the present study, the authors suggest diagnosing dentigerous cysts in the studied sample due to the presence of a well-circumscribed, unilocular radiolucency around the crown of an impacted or developing tooth, with their largest dimension ranging from 2.0 cm to 4.0 cm of diameter. However, to confirm a cystic lesion presence, complementary examinations, such as three-dimensional (3D) computed tomography (CT), biopsy, and cytopathologic analysis are recommended.^22-24^

Generally, dentigerous cysts are asymptomatic and they are usually diagnosed during routine dental care. However, they can become large in some cases, leading to tooth malposition or even causing tooth and bone resorption. The most frequently suggested treatment is the complete surgical enucleation and extraction of the associated impacted tooth or, in some cases, decompression or marsupialization in a developing tooth.^25^

Due to some physical or mental impairment inherent to MPS disease, clinicians may face some difficulties in conducting regular dental treatment in these individuals. In some cases, general anesthesia – to perform dental surgical procedures – may be necessary. However, dental practitioners should be aware that airway problems are common in MPS individuals, and the risks imposed during perioperative intubation should be considered. Therefore, in individuals with MPS, preventive and conservative dental treatments should be a priority to avoid some possible systemic injuries.^26^

In this study, increased condylar hypoplasia prevalence was observed among all MPS individuals compared to non-MPS individuals. One of the most explicit characteristics of MPS is limited joint mobility and ligamentous laxity due to the involvement of GAGs throughout the ligaments and joint capsules. The constant accumulation of GAGs in those regions can, consequently, lead to joint degeneration.^4^ Using CT views and 3D images, Torres et al. (2018) compared, in a case report, the mandibles of one MPS individual and one non-MPS patient. The authors observed the presence of higher coronoid process and condylar hypoplasia in the mandible of MPS individual, and this finding correlates with our observations.^28^

The progressive resorption of condyles may lead to several consequences for MPS individuals. Those alterations might provoke issues in articulating the mandible, resulting in an open bite as well as mandibular and facial asymmetry. Another factor is the condylar hypoplasia, which may also hinder the dental treatment, as some MPS patients can develop limitations on mouth opening. According to Chouinard, Kaban, Peacock^29^ (2018), it is important to perform an early diagnosis of temporomandibular joint abnormalities to determine the progression of the deformities, predict the prognosis, and plan an adequate treatment plan.^29^

In our study, it was impossible to carry out a complete analysis evaluating differences among individuals with different type of MPS regarding oral conditions, because of the low number of MPS individuals in each subgroup. Although systemic differences have been well recognized in the literature,^3^ the extent and significance of the differences among all MPS types concerning oral manifestations are still scarce. Most MPS individuals included in this study belonged to the group of MPS VI. MPS VI is described as the type that presents the most severe skeletal phenotype, although individuals usually do not have their neurological functions affected.^1^ Few studies have performed subgroup analyses to evaluate differences between MPS types. Yet, these studies have failed to compare all MPS types simultaneously due to the disease rare nature. Turra, Schwartz^30^ (2009) compared the differences among individuals with MPS types I, II, and VI regarding the structure of the stomatognathic system. Significant difference was found only for the position of the tongue between teeth. This outcome was more frequent in MPS VI. Sarmento, et al.^17^ (2015) evaluated MPS types I, IV, and VI and showed that all individuals with MPS type VI presented enlarged, cyst-like dental crypt. The presence of impacted tooth was also higher among individuals with MPS type VI.

Although previous studies have determined the presence of dental and/or maxillomandibular anomalies among MPS individuals, those studies were case series or cross-sectional studies with limited sample sizes that could not support strong statistical inferences. The presence of dental/maxillomandibular anomalies in MPS individuals cannot be defined or be associated with a severe or attenuated form of MPS, but the findings of our study can contribute to clinical practice with a better understanding of the clinical consequences that might be inherent in this rare disease. Some surgical interventions or orthodontic treatment might be necessary to correct or to minimize dental problems, and the clinicians must be aware of how to perform therapeutic interventions, considering the systemic and physical limitations that these individuals might present.

Some limitations of this study should be highlighted. The first is inherent to the design of a cross-sectional study. The results presented cannot be used to assess causality. They suggested an increased prevalence of dental alterations among MPS individuals. Secondly, a qualitative assessment of the presence and absence of dentofacial anomalies was performed. The authors suggest that future studies quantifying the extent of those alterations should be performed. Two-dimensional panoramic radiographs were used to measure dental and maxillomandibular alterations, and it is known that these exams present geometric distortion and structural superimposition. However, this study can be considered a starting point for future epidemiological studies regarding dental and maxillomandibular features in MPS individuals using a 3D tool. Finally, it is noteworthy that sample size should not be considered a major limitation as MPS has a relatively low overall prevalence, making large sample recruitment quite challenging.

## Conclusion

MPS individuals showed an increased frequency of dental and maxillomandibular incidental findings when compared to non-MPS individuals. The increased presence of supernumerary teeth, conoid teeth, taurodontism, impacted teeth, and root dilaceration was observed. The presence of bone rarefaction/furcation lesions, condylar hypoplasia, and radiolucent bone lesions were also more frequent among MPS individuals.
